# A MWCNTs-COOH/PSS nanocomposite–modified screen-printed electrode for the determination of synthetic phenolic antioxidants by HPLC with amperometric detection

**DOI:** 10.1007/s00604-022-05552-7

**Published:** 2022-11-24

**Authors:** Lucía Abad-Gil, Mayte García-Ríos, Carmen Isabel-Cabrera, M. Jesús Gismera, M. Teresa Sevilla, Jesús R. Procopio

**Affiliations:** Departamento de Química Analítica y Análisis Instrumental, Facultad de Ciencias, Universidad Autónoma de Madrid, Avda. Francisco Tomás y Valiente , 7. E-28049 Madrid, Spain

**Keywords:** Synthetic phenolic antioxidants, Carbon nanotubes, Polystyrene sulfonate, Nanocomposites, Electrochemical detection, HPLC

## Abstract

New sensing platforms based on screen-printed carbon electrodes modified with composites based on polystyrene sulfonate and oxidized multi-walled carbon nanotubes (PSS/MWCNTs-COOH/SPCE) have been used to develop a novel HPLC method with electrochemical detection (ECD) for the determination of the most used synthetic phenolic antioxidants in cosmetics: butylhydroxytoluene (BHT), butylhydroxyanisole (BHA), tert-butylhydroquinone (TBHQ) and propyl gallate (PG). Optimal separation conditions were achieved using methanol: 0.10 mol L^−1^ acetate solution at pH 6 as mobile phase with a gradient elution program from 60 to 90% of methanol percentage in 15 min. The electrochemical detection was carried out in amperometric mode using the PSS/MWCNTs-COOH/SPCE at + 0.80 V vs. Ag. Under these optimal separation and detection conditions, the limits of detection (LOD) were between 0.11 and 0.25 mg L^−1^. These LOD values were better, especially for BHT, than those previously published in other HPLC methods. Linear ranges from 0.37 mg L^−1^, 0.83 mg L^−1^, 0.69 mg L^−1^ and 0.56 mg L^−1^ to 10 mg L^−1^ were obtained for PG, TBHQ, BHA and BHT, respectively. RSD values equal or lower than 5% and 8% were achieved for repeatability and reproducibility, respectively. The HPLC-ECD method was successfully applied to analyze different cosmetic samples. Recovery values within 83–109% were obtained in the validation studies.

**Graphical Abstract:**

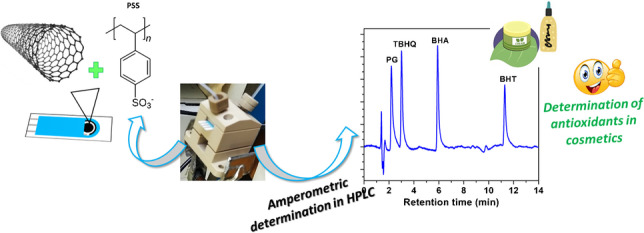

**Supplementary Information:**

The online version contains supplementary material available at 10.1007/s00604-022-05552-7.

## Introduction

**Fig. 1:**
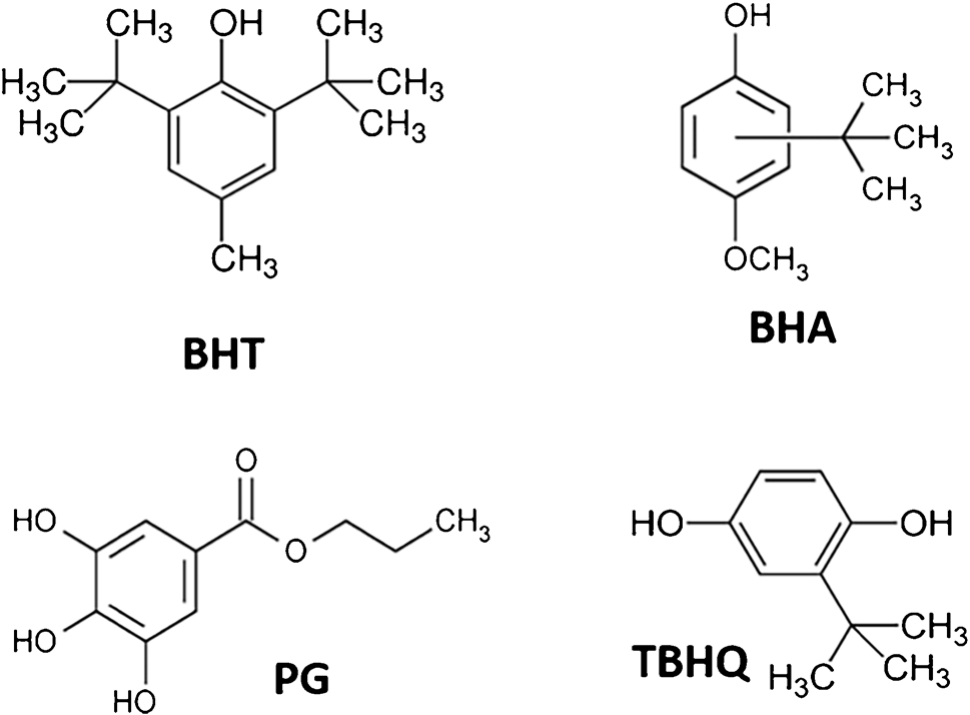
Chemical structures of BHT, BHA, PG and TBHQ

Cosmetic formulations include a wide variety of ingredients such as natural fats or oils that can be easily degraded by exposure to oxygen causing undesirable odours and other instabilities. The addition of antioxidants to these formulations is mandatory to avoid the rancidity of the products, length their shelf life and ensure consumers’ health. Although natural antioxidants such as citric acid or polyphenols can be used as preservation system in personal care products, synthetic phenolic antioxidants (SPAs) are more used for long-term preservation due to their low cost and better performance [1, 2]. Butylhydroxytoluene (BHT), butylhydroxyanisole (BHA), tert-butylhydroquinone (TBHQ) and propyl gallate (PG) (Fig. 1) are the most used SPAs in cosmetics [1, 3]. These products usually contain mixtures of these SPAs since the synergetic effects between these additives enhance the antioxidant power [4]. The overuse of SPAs may result in potential human health risks due to-carcinogenic, estrogenic and genotoxic activity of these compounds [5–10]. In addition, BHA, TBHQ, PG and BHT are well-known as skin sensitizers and can cause allergic contact dermatitis [11]. Despite the human health risks, the use of SPAs in Europe is not restricted in cosmetic products. Due to the increase in the concern about SPAs, the Scientific Committee on Consumer Safety (SCCS) has created in 2021 a working group to obtain data related to potential endocrine-disrupting properties of BHT and regulate the concentration of this compound in cosmetics [12]. The SCCS is also looking for scientific information of BHA due to its potential endocrine-disrupting properties [13].

Taking into account the potential risks of SPAs, the increase in the concern of the SCCS and their widespread use in cosmetics is necessary to develop analytical methods to determine BHT, BHA, PG and TBHQ in these products. The main challenge is to improve the determination of BHT, the most added antioxidant in cosmetics, for an adequate control of this compound. For this purpose, methods with better limits of detection are demanded. According to the complexity of cosmetic matrices, a sample preparation step is required prior the determination. Extraction methods such as solid-phase microextraction, ultrasound-assisted extraction or cloud-point extraction have been developed for the extraction of SPAs from cosmetics [14–16]. The analysis of SPAs in personal care products is usually accomplished together with antimicrobial preservatives [4, 15, 17] being separation techniques such as liquid chromatography [4], gas chromatography [17] or micellar electrokinetic chromatography [15] the most used. Among them, liquid chromatography coupled to diode-array (DAD) or mass spectrometer (MS) detectors is the main method for the identification and quantification of BHT, BHA, TBHQ and PG in cosmetics [4], and other products as fuels [18] or foods [19]. Electrochemical methods based on electrodes modified with different nanomaterials such as gold nanoparticles, carbon nanotubes (CNTs) or nanocomposites formed by CoSe_2_ nanoparticles and graphene have been developed for the determination of these compounds in biodiesel and food samples [20–22]. Among carbon nanomaterials used to develop electrochemical sensors, CNTs have received increasing attention due to their large surface area, high electrical conductivity, good mechanical strength and good chemical stability. CNTs tend to form aggregates due their high surface energy and the stabilization by π-π interactions among the tubes [23]. This behaviour along with their low solubility in polar solvents limits their applications. These drawbacks can be overcome by the modification of the nanomaterials. The most common procedure is the oxidation with an acid such as nitric acid, creating carboxylic groups onto the ends and sidewalls of CNTs [24]. Electrochemical techniques are an attractive option as detection system for in-flow determinations due to their sensitivity, selectivity, low cost and simpler use. In the last years, modified electrodes have been used for amperometric detection achieving better analytical properties such as lower limits of detection [25]. Despite these advantages, the electrochemical detection (ECD) used in chromatographic methods to determine BHT, BHA, TBHQ and PG is scarce [26–29].

The aim of this work was to develop an electrochemical sensor for the amperometric and sensitive determination of BHA, BHT, TBHQ and PG in HPLC systems. For this purpose, screen-printed carbon electrodes (SPCEs) were modified with nanocomposites formed by oxidized CNTs and the polymer polystyrene sulfonate (PSS). Previous studies carried out in our laboratory have demonstrated the good properties of PSS to get an adequate dispersion of different carbon nanomaterials [30]. The PSS/CNTs-COOH-based sensor has been used to develop an HPLC-ECD method for the simultaneous determination of the four SPAs in cosmetics. To the best of our knowledge, this is the first CNTs-based sensor used as electrochemical detector for the HPLC determination of BHA, BHT, PG and TBHQ in personal care products.

## Experimental section

### Reagents

All reagents used throughout this work were analytical grade and used with any further purification. PG (≥ 98%), BHT (≥ 99%), BHA (≥ 98.5%) and TBHQ (≥ 97%) were acquired from Sigma-Aldrich (https://www.sigmaaldrich.com). Standard stock solutions of each antioxidant (1000 mg L^−1^) were prepared in methanol (HPLC-grade, https://www.scharlab.com/) and stored in the darkness under refrigeration for a month. For HPLC analysis, working solutions were daily prepared by the adequate dilution of the stock solution of each antioxidant in the mobile phase. A 1.0 mol L^−1^ Britton-Robinson (BR) buffer solution prepared by mixing 0.40 mol L^−1^ glacial acetic acid (≥ 99.8%, Panreac, https://www.itwreagents.com), 0.40 mol L^−1^ boric acid (≥ 99.8%, Scharlab) and 0.40 mol L^−1^ phosphoric acid (85%, Scharlab) was used as supporting electrolyte in the electrochemical studies. The pH of the solution was adjusted by adding the adequate volume of 1.0 mol L^−1^ NaOH (Scharlab).

MWCNTs (10 nm of diameter and 1–2 μm in length) were from DropSens (https://www.dropsens.com) (ref DRP-MWCNTs) and PSS, as poly(4-styrenesulfonic acid) sodium salt (average MW 70,000) was purchased from Sigma-Aldrich. Nitric acid (65%) used to oxidize MWCNTs was acquired from Scharlab. Potassium ferrocyanide (K_4_[Fe(CN)_6_]0.3H_2_O) (≥ 99%, Panreac), hexaamineruthenium (III) chloride (≥ 98%, Sigma-Aldrich) and potassium chloride (≥ 99.5%, Sigma-Aldrich) were used for the characterization of the modified electrodes.

Ultrapure water (Type I) obtained from a Mili-RO-Milli-Q water system (https://www.merckmillipore.com) was used throughout this work.

### Instrumentation

Electrochemical experiments were carried out using the μSTAT 400 bipotentiostat (DropSens) controlled by the “Drop-View 8400” software. SPCEs (ref. DRP-110, Metrohm DropSens) formed by a carbon ink as working electrode (4 mm diameter), Ag as pseudo-reference electrode and carbon ink as counter electrode were used for voltammetric measurements and amperometric detection. Specific connectors (DRP-CAST and DRP-DSC, DropSens) were used to connect the SPCEs to the potentiostat. Electrochemical impedance spectroscopy (EIS) measurements were acquired from a EcoChemie BV µAutolab FRA 2 type III controlled by the Frequency Response Analyser (FRA) software. The frequency range from 10 kHz to 0.1 Hz with a sinusoidal voltage perturbation of 10 mV of amplitude was used.

HPLC measurements were performed using a Jasco™ liquid chromatography system formed by a Quaternary Gradient Pump (Jasco, PU-2089 plus, https://www.jasco-spain.com/) equipped with a vacuum degasser, an auto-injector (Jasco, AS-2055 plus), a column oven (Jasco, CU-2067 plus) and a multiwavelength detector (Jasco, MD-2010 plus). For chromatographic data acquisition and handling, a LC-NetII/ADC interface with the Jasco ChromPass chromatography data system was used. A μAutolab potentiostat (EcoChemie, https://www.metrohm.com) controlled by the software General Purpose Electrochemical System (GPES Manager 4.9007 version) was used for amperometric detection.

A Flow Injection System (FIA) consisted in a Gilson pump (model 302C, https://es.gilson.com/), a Rheodyne 7125 injection valve provided with a 20 μL sample loop and the bipotentiostat μSTAT 200 controlled by the Drop-View software was used to carry out the hydrodynamic voltammetric studies.

A flow cell (HPLCELL, Metrohm) adapted to SPCEs was used for both FIA and HPLC measurements.

### Preparation of the modified electrodes

Prior to modification of the working electrode surface, MWCNTs were oxidized to incorporate carboxylic groups in their structure and thus improve their polarity following the procedure described in the literature [31]. In short, MWCNTs were suspended in 5.0 mol L^−1^ of nitric acid under mechanical stirring, for 24 h. The solid product was filtered and washed with ultrapure water until neutral pH. The obtained MWCNTs-COOH was dried 24 h in an oven at 80 °C.

#### Preparation of PSS/MWCNTs-COOH suspension

The PSS/MWCNTs-COOH suspension was prepared by mixing the adequate amount of MWCNTs-COOH in a 3% PSS aqueous solution to obtain 1% MWCNTs-COOH suspension. The mixture was sonicated in an ultrasonic bath (model P30HS Elmasonic, https://www.elma-ultrasonic.com/) at 80 kHz and 100 W at 25 °C for 3 h to disperse the CNTs. The obtained PSS/MWCNTs-COOH suspension was stored under refrigeration at 4 °C until use. For comparison purposes, a 1% suspension of non-oxidized MWCNTs in PSS (PSS/MWCNTs) was prepared following the procedure described above.

#### Preparation of the nanocomposite-based sensors

Before electrode modification, SPCEs were activated in KCl 0.10 mol L^−1^ to remove any impurity and obtain a reproducible electrode surface. For this purpose, 20 cyclic scans were recorded between − 0.50 and + 0.90 V at 0.500 V s^−1^ scan rate.

The working electrode surface was modified by a drop-casting method putting 4 μL of the PSS/MWCNTs-COOH suspension onto the electrode and dried overnight at room temperature. The sensors obtained were denoted as PSS/MWCNTs-COOH/SPCE. The modified electrodes can be stored at room temperature for at least 3 months without changes in analytical performance.

### Characterization of the electrode surfaces

The morphology of the surface of the unmodified and modified SPCEs was characterized by scanning electron microscopy (SEM) obtaining images at 300,000 × magnification (NOVA NanoSEM230, FEI Company, https://www.thermofisher.com). To carry out these studies, the working electrode was metalized by sputtering gold (Leica EM ACE200, Leica Microsystems, https://www.leica-microsystems.com).

The electrode surface of the sensors was electrochemically characterized by recording cyclic voltammograms of 0.0010 mol L^−1^ [Fe(CN)_6_]^4−^ and [Ru(NH_3_)_6_]^3+^ in 0.10 mol L^−1^ KCl aqueous solutions from − 0.35 V to + 1.00 V and − 0.50 V to + 0.30 V, respectively, at 0.050 V s^−1^ scan rate. To perform the EIS characterization, a 0.0010 mol L^−1^ [Fe(CN)_6_]^4−^ in 0.10 mol L^−1^ KCl solution was used as redox probe and Open Circuit Potential (OCP) was applied.

### Electrochemical measurement procedures

The electrochemical response of the antioxidants at 200 mg L^−1^concentration on the unmodified and modified SPCEs was evaluated by linear sweep voltammetry at 0.100 V s^−1^ scan rate in a mixture 50:50 (v/v) methanol: 0.10 mol L^−1^ BR buffer solution. Between measurements, the electrode surface of the unmodified and modified sensors was rinsed with methanol and then five cyclic scans were performed in the supporting electrolyte. Following this procedure, a reproducible electrode surface is obtained, and the electrode can be repeatedly used for about 15 times. All electrochemical experiments were carried out at room temperature. For the measurement, 60 µL of the solution was pipetted and put onto the electrodes.

### Chromatographic conditions

Separations were carried out using a Kromaphase 5 μm C_18_ (150 mm × 4.6 mm) column (Scharlab) thermostated at 40 °C. The injection volume was 20 μL. The mobile phase consisted in 0.10 mol L^−1^ sodium acetate solution at pH 6, previously vacuum filtered throughout a 0.45-μm nylon filter, and methanol as organic modifier at 1.0 mL min^−1^ flow rate. To obtain a suitable separation of the antioxidants, a gradient elution mode was used. It consisted in a linear gradient from 60 to 80% methanol in 5 min, followed by an increase in methanol percentage up to 90% in 2 min, to finally maintain this percentage for 5 min. Then, 60% methanol was introduced for 4 min to stabilize the system before the next injection. The amperometric detection of the antioxidants was carried out on PSS/MWCNTs-COOH/SPCE devices and applying + 0.80 V (vs. Ag) as working potential. Prior the HPLC measurements, PSS/MWCNTs-COOH/SPCE platforms were treated by applying the working potential for 2 min to activate and obtain a reproducible electrode surface.

### Sample preparation

Commercial cosmetic products, such as a micellar water and a moisturizing cream, were analyzed using the developed HPLC method with amperometric detection. According to the label, the studied cosmetic samples contained at least one of the assayed antioxidants. For the liquid aqueous–based sample, a simple dilution with mobile phase was required before HPLC analysis. Antioxidants were extracted from the moisturizing cream using the following ultrasound-assisted extraction (UAE) method [32]: 1.000 g of sample was weighted, and 9 mL of methanol was added. Then, the mixture was sonicated for 10 min at 25 °C, using 80 kHz and 100 W. The supernatant was separated by centrifugation at 2500 rpm for 5 min. No additional dilution was required for HPLC analysis.

Prior to HPLC measurements, all samples were filtered using a 0.45-μm nylon filter.

## Results and discussion

### Characterization of the electrodes

#### Scanning electron microscopy

The stability of the PSS/MWCNTs and PSS/MWCNTs-COOH suspensions was first evaluated. A homogeneous and stable dispersion was obtained using oxidized MWCNTs since the incorporation of carboxylic groups increases the polarity of the nanomaterial, whereas the suspension prepared with non-oxidized-MWCNTs was not homogeneous and stable along time (Figure S1 in Electronic Supplementary Material, ESM). In fact, the PSS/MWCNTs-COOH suspension presented very good stability, with an appearance of a black ink, even 1 year after its preparation.

**Fig. 2:**
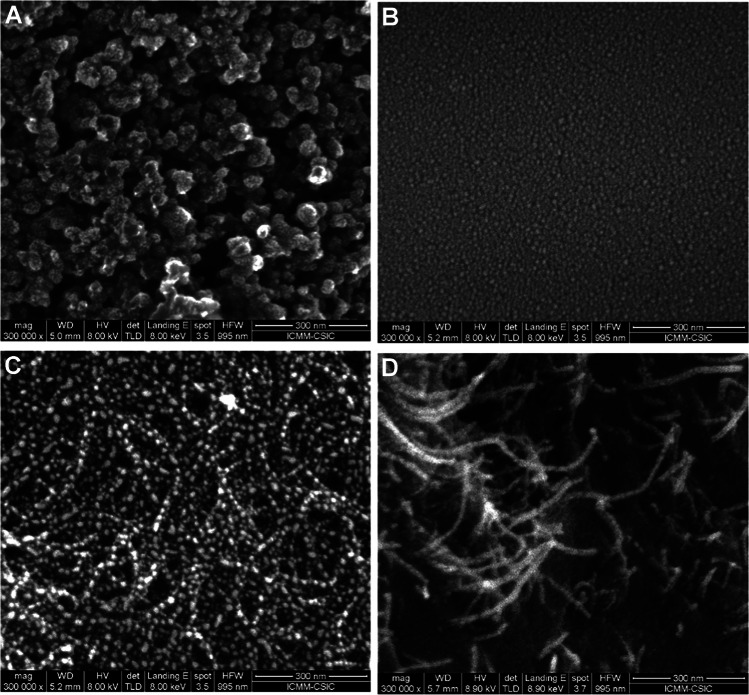
SEM images of the working electrode surface of **A** SPCE, **B** PSS/SPCE and **C** PSS/MWCNTs-COOH/SPCE and D PSS/MWCNTs/SPCE

The morphology of the unmodified SPCE and the PSS/MWCNTs-COOH/SPCE electrodes was studied by SEM. For comparison purposes, the surface of a SPCE modified with PSS (PSS/SPCE) and with PSS/MWCNTs was also characterized. As can be seen in the SEM images (Fig. 2), the surfaces of the SPCE, PSS/SPCE, PSS/MWCNTs-COOH/SPCE and PSS/MWCNTs/SPCE electrodes present different morphologies. A more homogeneous and smooth electrode surface was observed when the working electrode was modified with PSS compared to the unmodified SPCE (Fig. 2A and B). As can be seen in Fig. 2C and D, MWCNTs are entrapped in the polymer network and distributed on the surface. Comparing the electrodes modified with the different suspensions formed by PSS and MWCNTs or MWCNTs-COOH, a more homogeneous distribution of the CNTs was observed when the nanomaterial was treated with nitric acid. This was expected since PSS/MWCNTs suspensions are not stable. Thus, PSS/MWCNTs-COOH suspensions should be used to obtain homogeneous electrode working surfaces.

#### Electrochemical characterization

The electrode surface of SPCE, PSS/SPCE and PSS/MWCNTs-COOH/SPCE was electrochemically characterized by cyclic voltammetry using 0.0010 mol L^−1^ ferrocyanide and hexaamineruthenium (III) in 0.10 mol L^−1^ KCl as electrochemical probes. The measurements were carried out by triplicate in different electrodes. Table 1 shows the electroactive area of the electrodes calculated using the Randles–Sevcik equation:

**Table 1:** Electroactive area (*A*) and parameters calculated from the cyclic voltammograms of 0.0010 mol L^−1^ [Fe(CN)_6_]^4−^ and [Ru(NH_3_)_6_]^3+^ in 0.10 mol L^−1^ KCl registered in the different electrodes. Geometric area, *A*_*g*_ = 0.126 cm^2^ (*n* = 3)

Electrode	Electrochemical probe	*A* (cm^2^)	*A/A*_*g*_	*I*_*pa*_/*I*_*pc*_	Δ*E* (V)
SPCE	[Fe(CN)_6_]^4−^	0.079 ± 0.003	0.63	0.95 ± 0.02	0.17 ± 0.03
	[Ru(NH_3_)_6_]^3+^	0.071 ± 0.003	0.56	0.93 ± 0.03	0.073 ± 0.006
PSS/SPCE	[Fe(CN)_6_]^4−^	0.060 ± 0.003	0.48	0.9 ± 0.1	0.25 ± 0.06
	[Ru(NH_3_)_6_]^3+^	0.13 ± 0.01	1.05	0.93 ± 0.02	0.048 ± 0.006
PSS/MWCNTs-COOH/SPCE	[Fe(CN)_6_]^4−^	0.13 ± 0.01	1.03	0.96 ± 0.01	0.097 ± 0.001
	[Ru(NH_3_)_6_]^3+^	0.23 ± 0.01	1.79	0.99 ± 0.02	0.054 ± 0.003

$$I_p=\left(2.69\times10^5\right)n^\frac32{ACD}^\frac12\upsilon^\frac12$$

In this equation, *I*_*p*_ is the anodic peak current (A), *n* is the number of electrons transferred in the redox process, *A* is the electroactive area (cm^2^), *C* is the bulk concentration (mol cm^−3^), *D* is the diffusion coefficient (6.3 $\times$ 10^−6^ cm^2^ s^−1^ for ferrocyanide and 8.43 $\times$ 10^−6^ cm^2^ s^−1^ for hexaamineruthenium (III)) and *υ* is the scan rate (V s^−1^). Table 1 also includes the ratio of anodic to cathodic peak currents (*I*_*pa*_/*I*_*pc*_), the separation between the anodic and cathodic peak potentials (Δ*E*) and the ratios of electroactive to geometric area (*A/A*_*g*_) obtained using both electrochemical probes. As can be seen, for PSS/SPCE and PSS/MWCNTs-COOH/SPCE platforms, different values of the electroactive area and *A/A*_*g*_ were obtained depending on the electrochemical probe. This can be explained by the different electrostatic interactions that are established between the redox probe and the composite, e.g. on PSS/SPCE electrodes, a lower electroactive area was observed using the negative probe than with the positive one due to the repulsion that occurs between the negatively charged sulfonate groups of PSS polymer and [Fe(CN)_6_]^4−/3−^. For both redox probes, the highest electroactive area was observed on PSS/MWCNTs-COOH/SPCE platforms due to the excellent properties of MWCNTs. As might be expected, the electroactive area on PSS/MWCNTs-COOH/SPCE electrode with Ru(NH_3_)_6_]^3+^ was higher than the obtained value using [Fe(CN)_6_]^4−^ as redox probe (Table 1). The *A/A*_*g*_ ratio indicates the real active area of the electrode, obtaining the best results using PSS/MWCNTs-COOH/SPCE electrodes. The peak current ratio (*I*_*pa*_/*I*_*pc*_) is close to 1 for all sensors. A higher peak potential difference (Δ*E*) is observed for all electrodes than that expected for the [Fe(CN)_6_]^4−/3−^ reversible system that involves the transfer of one electron. For the characterization with [Ru(NH_3_)_6_]^3+^, Δ*E* values close to 0.060 V were observed for all electrodes.

**Fig. 3:**
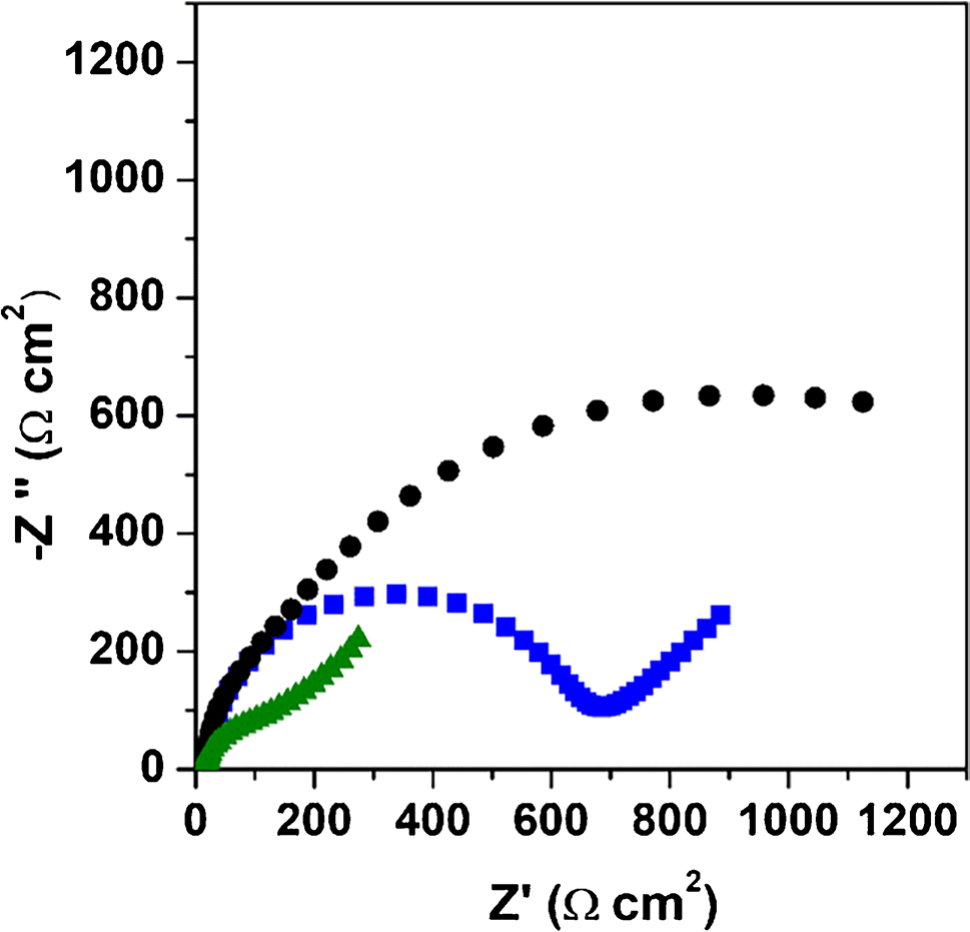
Complex plane impedance spectra obtained for SPCE (■), PSS/SPCE (●) and PSS/MWCNTs-COOH/SPCE (▲) in a 1.00 mmol L^−1^ [Fe(CN)_6_]^4−/3−^ and 0.10 mol L^−1^ KCl solution. Experimental conditions: open circuit potential, 10 mV of amplitude and frequency range from 10 kHz to 0.10 Hz

To obtain information about the electrical interfacial properties and charge transfer processes, the Nyquist plots of the SPCE, PSS/SPCE and PSS/MWCNTs-COOH/SPCE platforms were registered. EIS measurements were performed by applying OCP and using 0.0010 mol L^−1^ [Fe(CN)_6_]^4−^ in 0.10 mol L^−1^ KCl as redox probe. Figure 3 shows the Nyquist plots of the different tested platforms. As can be seen, different profiles were obtained for each electrochemical platform. Three regions for PSS/SPCE and PSS/MWCNTs-COOH/SPCE and two regions for SPCE can be differentiated in the spectra, in all cases observing a small semicircle at high frequencies that corresponds to the electron transfer process. For SPCE at medium and low frequencies and for PSS/SPCE and PSS/MWCNTs-COOH/SPCE at medium frequencies, a linear part related to diffusional phenomenon was observed. At low frequencies for the PSS/SPCE, a semicircle with larger diameter attributed to charge separation/transport on the modified electrode and the solution was observed. For PSS/MWCNTs-COOH/SPCE, a linear part was observed at the low frequency region correlated with a charge separation phenomenon. Different values of resistance and constant phase element were observed on SPCE, PSS/SPCE and PSS/MWCNTs-COOH/SPCE, indicating the influence of the presence of the composite in the charge separation of the electrode/modifier interface. An increase of the constant phase element and a decrease in the resistance were observed on the electrode modified with the PSS/MWCNTs-COOH composite, suggesting an easier electron transfer and a greater charge separation. The increase of constant phase element can be explained by the higher electroactive surface area observed when the MWCNTs-COOH was incorporated in the composite. These results are in accordance with those obtained in the characterization by cyclic voltammetry.

### Electrochemical behaviour of SPAs on modified SPCE

**Fig. 4:**
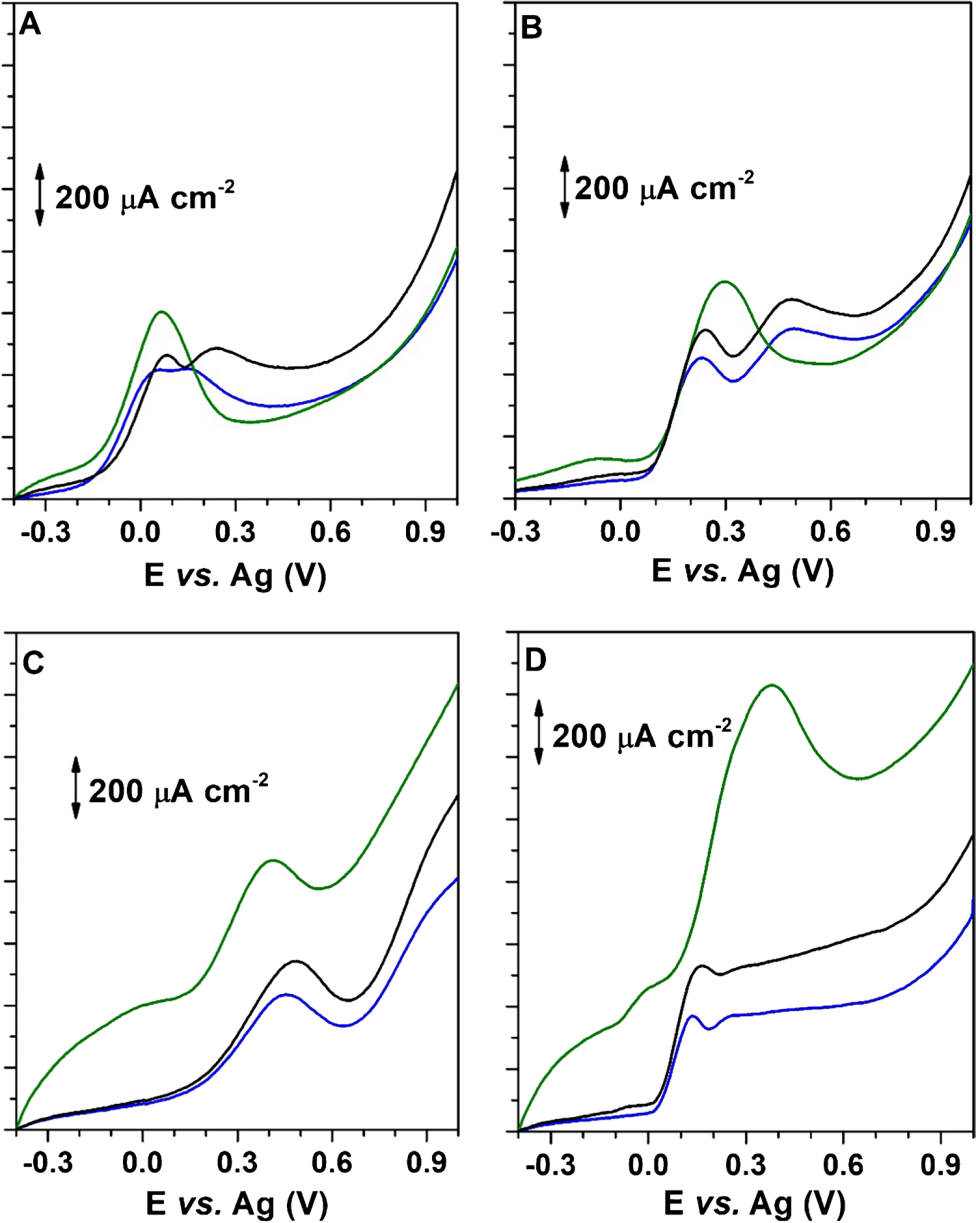
Linear sweep voltammograms of 200 mg L^−1^ of **A** TBHQ, **B** BHA, **C** BHT and **D** PG in 0.10 mol L^−1^ BR buffer solution at pH 6 in the presence of 50% of methanol on different SPCE: SPCE (—), PSS/SPCE (—) and PSS/MWCNTs-COOH/SPCE (—). Scan rate: 0.100 V s^−1^

The electrochemical response of the studied SPAs was evaluated on SPCE, PSS/SPCE and PSS/MWCNTs-COOH/SPCE using linear sweep voltammetry in 0.10 mol L^−1^ BR buffer at pH 6 containing 50% of methanol. The LS voltammograms obtained are shown in Fig. 4. As can be seen, all SPAs are electroactive on the unmodified SPCE, observing for TBHQ, PG and BHA two anodic peaks, poorly defined for TBHQ and especially for PG. A well-defined anodic peak at + 0.45 V is observed for BHT. As can be seen in Fig. 4, an increase in the response of all SPAs is observed using PSS/SPCE versus SPCE. Moreover, a greater increase is observed on PSS/MWCNTs-COOH/SPCE. This is expected due to the absorptive and catalytic characteristics of CNTs. Taking into account the obtained results, PSS/MWCNTs-COOH/SPCE platforms were selected for the amperometric detection of antioxidants to achieve the maximum sensitivity in the measurement. The electrochemical behaviour of SPAs was also evaluated at different pH values. No significant differences were observed on the response of SPAs, so pH 6 was selected for the following studies.

### Optimization of chromatographic conditions

The separation conditions of PG, TBHQ, BHA and BHT were optimized using 10 mg L^−1^ multi-analyte standard solutions and using DA as detection system. A wavelength of 280 nm was selected for the simultaneous determination of SPAs [33]. Percentages of methanol between 70 and 95% in 0.10 mol L^−1^ acetate solution at pH 6 were evaluated to establish the optimal composition of the mobile phase. Long analysis times were observed using low methanol percentages and peak resolutions below 1.5 were found when high percentages of methanol were used in isocratic mode. According to these results, a gradient elution mode was optimized for the separation of the four SPAs. Adequate analysis times and resolutions were observed when the following gradient of methanol was used: two successive linear gradients from 60 to 80% in 5 min and from 80 to 90% in 2 min, then an isocratic elution for 5 min and finally a linear gradient to the initial conditions (60%) in 30 s. Before the next injection, the system was stabilized for 2.5 min under the initial conditions. Using this gradient elution program, the retention time values of PG, TBHQ, BHA and BHT were 2.25 ± 0.04, 3.11 ± 0.06, 6.05 ± 0.08 and 11.43 ± 0.08 min, respectively, obtaining a reproducibility of the retention times (*n* = *3*) between 0.7 and 2%. Under the optimized conditions, the separation of the four SPAs can be achieved in less than 12 min, being 15 min the total analysis time including the equilibration time.

### Optimization of the electrochemical detection

**Fig. 5:**
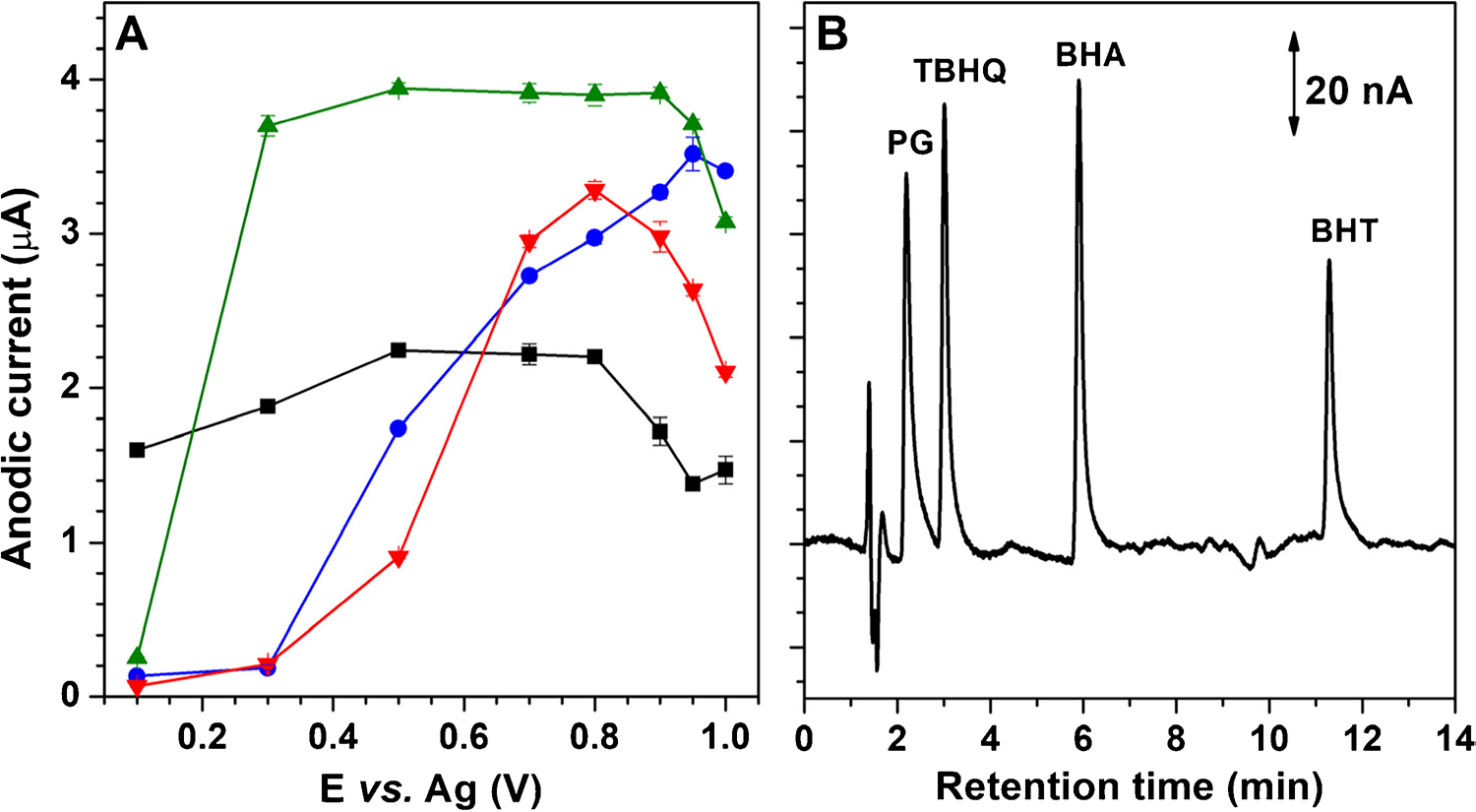
**A** Hydrodynamic voltammograms of PG (■), TBHQ (●), BHA (▲) and BHT (▼) at 10 mg L^−1^ concentration (*n* = 3) using 0.10 mol L^−1^ pH 6 acetate solution containing 85% methanol at 1.0 mL min^−1^ flow rate as carrier. **B** Chromatogram obtained for 5.0 mg L^−1^ multi-analyte solution of SPA using the PSS/MWCNTs-COOH/SPCE electrode in the ECD at + 0.80 V vs. Ag

Hydrodynamic voltammograms of SPAs were obtained to select the most suitable working potential. For this purpose, the electrochemical response of PG, TBHQ, BHA and BHT was evaluated on PSS/MWCNTs-COOH/SPCE using the FIA system described in the “Experimental section” and applying a potential range between + 0.10 V and + 1.0 V. A 0.10 mol L^−1^ acetate solution at pH 6 containing 85% methanol was used as carrier. Figure 5A shows the hydrodynamic voltammograms obtained. As can be seen, anodic current of TBHQ and BHT increases as the working potential increases, achieving the maximum current at + 0.95 V for TBHQ and + 0.80 V for BHT. On the other hand, BHA and PG signals remain constant from + 0.30 V to + 0.90 V and from + 0.50 V to + 0.80 V, respectively. According to these results, + 0.80 V was chosen as working potential for the amperometric determination of PG, TBHQ, BHA and BHT.

To evaluate the stability of the PSS/MWCNTs-COOH/SPCE platforms, a repeatability study was carried out in the FIA system under the same conditions as above and applying + 0.80 V as working potential. For this purpose, a 10 mg L^−1^ BHA solution was injected 24 times. No significant changes were observed on FIA signals for successive injections, obtaining good repeatability (5% in terms of relative standard deviation, RSD, *n* = 24). These results demonstrate the suitability of PSS/MWCNTs-COOH/SPCE devices to be used as electrochemical detectors for the HPLC determination of PG, TBHQ, BHA and BHT.

Figure 5B shows the typical chromatogram obtained using the optimal separation and detection (+ 0.80 V vs. Ag on PSS/MWCNTs-COOH/SPCE) conditions.

### Analytical parameters of the HPLC-ECD method

The calibration plots of PG, TBHQ, BHA and BHT were obtained using the proposed HPLC-ECD method (Figure S2 in ESM). For this purpose, multi-analyte standard solutions in concentrations ranging between 0.10 and 13 mg L^−1^ were injected in triplicate in the HPLC system. The principal analytical parameters obtained for the SPAs are shown in Table 2. For all SPAs, a strong relationship between peak area and concentration (*r* > 0.9990) was observed in the studied concentration range. The highest sensitivity, expressed as the slope of the calibration plot, was observed for BHA. The intercept values of all SPAs were statically equal to zero (95% confidence level). The limits of detection (LOD) and quantification (LOQ) were calculated using a signal-to-background noise ratio of 3:1 and 10:1, respectively, from the standard deviation of the peak area for a PG, TBHQ, BHA or BHT standard solution close to the limit of detection. As can be seen, LODs lower than and equal to 0.25 mg L^−1^ were obtained for all antioxidants using the HPLC-ECD method. The repeatability and reproducibility of the method were calculated by injecting 5.0 mg L^−1^ multi-analyte solution in the same day (*n* = 3) and in different days (*n* = 3), respectively. RSD values in terms of repeatability were lower or equal to 5%, and below or equal to 8% in terms of reproducibility using the same electrode. The reproducibility of the modification was evaluated by injecting 5.0 mg L^−1^ multi-analyte solution and using different electrodes (*n* = 3). Good values of reproducibility (RSD values below 10%) were obtained demonstrating the suitability of the modification process performed.

**Table 2:** Analytical parameters of PG, TBHQ, BHA and BHT obtained using the HPLC-ECD method

Antioxidant	Sensitivity (nA·min·L·mg^−1^)	*r*	LOD (mg L^−1^)	LOQ (mg L^−1^)	Repeatability, % RSD^a^	Reproducibility, % RSD^a,b^
PG	2.59 ± 0.07	0.9992	0.11	0.37	3	4
TBHQ	3.03 ± 0.04	0.9997	0.25	0.83	4	8
BHA	3.35 ± 0.02	0.9999	0.21	0.69	2	8
BHT	2.20 ± 0.04	0.9995	0.17	0.56	5	6

^a^*n* = 3

^b^Different days, same electrode

Table 3 compares some features of SPAs determination using the proposed HPLC-ECD- PSS/MWCNTs-COOH/SPCE method with those obtained with other HPLC methods previously published. In general, the here reported LOD values for PG, TBHQ and BHA with the proposed HPLC-ECD method using PSS/MWCNTs-COOH/SPCE are comparable to those obtained by other HPLC or CE methods with ECD [26, 27, 29]. The LOD obtained for BHT using the developed method is between 6 and 10 times lower than those obtained using other HPLC-ECD methods [26, 28, 29]. The LOD values for PG, TBHQ, BHA and BHT are lower than those obtained using UV [33]. The very low LOD values reported by some published HPLC–UV methods [16, 19] are due to the preconcentration step performed as cloud-point extraction (CPE) [16] or polymer monolith microextraction (PMME) [19] getting enrichment factors between 11 and 53 [19]. The fluorescence detector [34], applying background correction and a second-order calibration method, allows to obtain low detection limits for TBHQ, BHA and BHT but the LOD for PG is higher than the found in the present work. The precision of the proposed method is comparable to that obtained by the HPLC-ECD method using a modified electrode [29], and by the HPLC–UV methods.

**Table 3:** Comparison of the proposed HPLC-ECD method with those previously published

Method	Electrode material	LOD (mg L^−1^)				% RSD				Reference
		PG	TBHQ	BHA	BHT	PG	TBHQ	BHA	BHT	
MECK-ECD	Carbon disc	0.06	0.13	0.18	0.66	0.6	0.8	2	2	[26]
CE-ECD^a^	Gold electrode	0.91	0.13	0.25	-	2	3	4	-	[27]
HPLC-ECD	GCE^b^	-	0.020	0.018	1.33	-	1	1	1.3	[28]
HPLC-ECD	Nickel phthalocyanine polymer/GCE^b^	0.15	-	0.11	0.60	5	-	7	8	[29]
HPLC–UV	-	0.3	0.5	0.5	0.5	< 4	< 4	< 4	< 4	[33]
HPLC-UV^c^	-	0.002	0.001	0.003	0.002	7	7	9	8	[19]
HPLC-UV^d^	-	0.014	0.085	0.019	0.043	5	4	3	4	[16]
HPLC-FLD	-	0.193	0.020	0.0097	0.0412	-	-	-	-	[34]
HPLC-ECD	PSS/MWCNTs-COOH/SPCE	0.11	0.25	0.21	0.17	4	8	8	6	This work

^a^Lab-chip based capillary electrophoresis-electrochemical detection system

^b^Glassy carbon electrode

^c^LOD values after a preconcentration process

^d^LOD values after a cloud-point extraction (CPE) process

### Analysis of real samples

The HPLC-ECD method using PSS/MWCNTs-COOH/SPCE as working electrode was used for the determination of PG, TBHQ, BHA and BHT in two different cosmetic samples: a micellar water and a moisturizing cream that, according to the label, contain BHT. As has been indicated in the “Experimental section,” micellar water was diluted with the mobile phase, whereas antioxidants were extracted from the moisturizing cream using the described UAE procedure. The analyses were performed in triplicate, and the concentration of antioxidants in the cosmetics was determined by an external standard calibration method. For validation purposes, the amount of the analytes in the samples was also calculated using the DAD. The obtained concentrations are shown in Table 4. As can be seen, BHT was found in both samples. According to the obtained results, no significant differences (with a 95% confidence level) were observed for both samples between the concentrations calculated using DAD and ECD. Using the ECD, TBHQ was found in the moisturizing cream (0.0019 ± 0.0002%, w/w) although this antioxidant was not detailed in the label. Due to the low selectivity of DAD, TBHQ cannot be detected using this detector since unidentified compounds present in the sample elute at the retention time of this SPA (Figure S3 in ESM). This fact shows the good selectivity of the proposed HPLC method using ECD with the PSS/MWCNTs-COOH/SPCE sensor.

**Table 4:** Concentration of the SPAs in the samples determined by the proposed HPLC method with the PSS/MWCNTs-COOH/SPCE electrode as ECD detector and using the DAD detector

Cosmetic sample	Concentration of SPAs, % (w/w) (mean value ± standard deviation, *n* = 3)							
	PG		TBHQ		BHA		BHT	
	HPLC-ECD	HPLC–DAD	HPLC-ECD	HPLC–DAD	HPLC-ECD	HPLC–DAD	HPLC-ECD	HPLC–DAD
Micellar water	n.i	n.i	<LOD	n.i	n.i	n.i	0.0018 ± 0.0002	0.0021 ± 0.0001
Moisturizing cream	n.i	n.i	0.0019 ± 0.0002	*	n.i	n.i.	0.0012 ± 0.0001	0.00125 ± 0.00008

*n.i. *not indicated in the label

*Unable detection

**Fig. 6:**
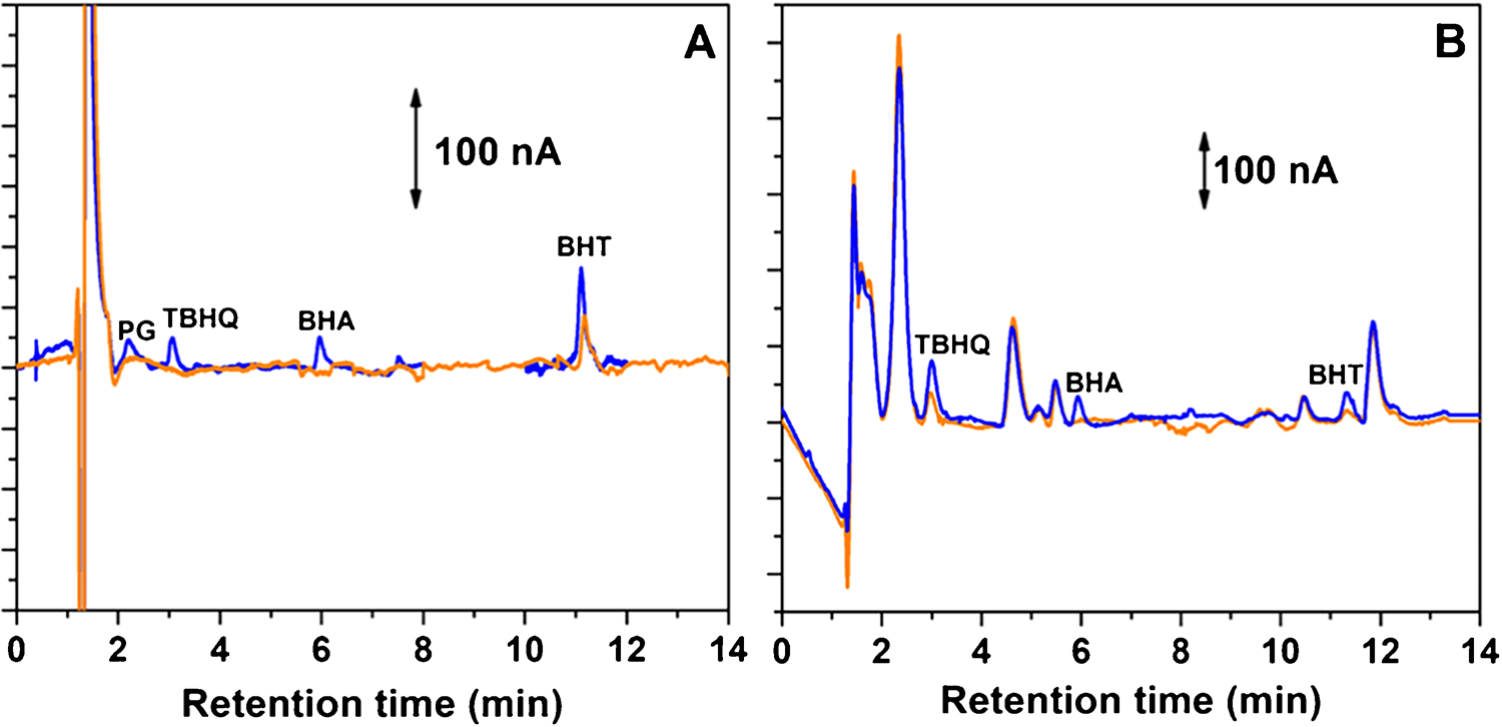
Chromatogram of **A** micellar water and **B** moisturizing cream obtained before (—) and after (—) fortification with PG, TBHQ, BHA and BHT, using the proposed HPLC-ECD method with the PSS/MWCNTs-COOH/SPCE electrode

The micellar water and the moisturizing cream were spiked with all the SPAs to validate the HPLC-ECD method. For this purpose, the samples were fortified in triplicate with the adequate volume of a multi-analyte standard solution of the antioxidants. Figure 6 shows the chromatograms obtained for both samples before and after their fortification. Recoveries from 87 to 109% and between 83 and 97% were obtained for all antioxidants in the micellar water and moisturizing cream, respectively, using the HPLC-ECD method (Table S1 in ESM). Except for PG in the moisturizing cream, the recovery values obtained in both samples were close to 100%, indicating the selectivity of the proposed HPLC-ECD method. As can be seen in Fig. 6B, PG cannot be detected with ECD or DAD due to the presence of an interfering compound. This compound was identified as one of the bioactive ingredients of the cream. The unable detection of PG in this fortified sample cannot be considered a critical point since BHT is the antioxidant usually added to these cosmetics. Considering these results, the proposed HPLC method with the PSS/MWCNTs-COOH/SPCE sensor for the amperometric detection can be considered a very suitable method to determine SPAs in different cosmetic samples.

## Conclusions

In the present work, a sensor based on the modification of a screen-printed carbon electrode (SPCE) with the nanocomposite formed by PSS and MWCNTs-COOH was developed and successfully used to determine by HPLC-ECD the most used synthetic phenolic antioxidants (SPAs) PG, TBHQ, BHA and BHT. An enhancement of the signal of the antioxidants was observed using the developed platforms compared to the obtained signal with unmodified SPCE. Using the PSS/MWCNTs-COOH/SPCE device, sensitive responses were obtained for the SPAs. In fact, a significant improvement was observed in the limit of detection of BHT compared to those reported in previous works on BHT determination. This is a very interesting advantage since BHT is the most used antioxidant for cosmetic preservation and the concern about the risks of this compound has increased in recent years. The proposed HPLC-ECD method was applied to analyze cosmetics with different matrices with successful results. For example, TBHQ was determined in the samples using the ECD whereas this antioxidant was not detected when DAD was used as detector of the chromatographic system. In addition, good recoveries were obtained in validation studies. These results demonstrate a better performance and suitability of the PSS/MWCNTs-COOH/SPCE device as ECD to determine SPAs in cosmetic products than the DAD. Thus, the proposed HPLC-ECD method can be used to control the amount of PG, TBHQ, BHA and BHT in cosmetics. The determination of PG could be interfered in some cosmetics due to the presence of some components. To analyze those samples, a more complex sample treatment can be used.

## Supplementary Information

Below is the link to the electronic supplementary material.

Supplementary file1 (PDF 400 KB)
