# Supplementary material for: A MWCNTs-COOH/PSS nanocomposite–modified screen-printed electrode for the determination of synthetic phenolic antioxidants by HPLC with amperometric detection
Source: Mikrochim Acta. 2022 Nov 24;189(12):469. doi: 10.1007/s00604-022-05552-7 (PMC9691489; doi:10.1007/s00604-022-05552-7)
Supplement: Supplementary file 1 — Supplementary file1 (PDF 400 KB) [file 604_2022_5552_MOESM1_ESM.pdf]

ELECTRONIC SUPPORTING MATERIAL

A MWCNTs-COOH/PSS nanocomposite modified screen-printed electrode for the determination of synthetic phenolic antioxidants by HPLC with amperometric detection

Lucía Abad-Gil (https://orcid.org/0000-0002-0296-8053), Mayte García-Ríos, Carmen Isabel-Cabrera, M. Jesús Gismera (https://orcid.org/0000-0002-6344-0148), M. Teresa Sevilla (https://orcid.org/0000-0003-0016-9714), Jesús R. Procopio (https://orcid.org/0000-0003-1200-8408)*

Departamento de Química Analítica y Análisis Instrumental, Facultad de Ciencias, Universidad Autónoma de Madrid. Avda. Francisco Tomás y Valiente, 7. E-28049 Madrid, Spain

* Corresponding author e-mail: lucia.abad@uam.es

Figure S1. Stability of the suspensions of oxidized- and non-oxidized- MWCNTs in PSS aqueous solutions

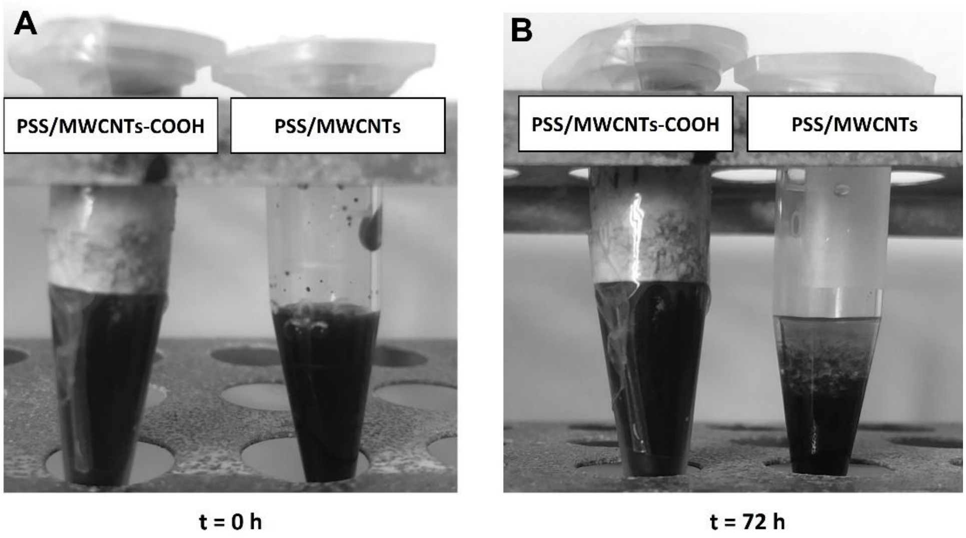

Figure S1. Photographs of PSS/MWCNTs-COOH and PSS/MWCNTs suspensions immediately after sonication (t =0) and 3 days later (t = 72h).

__Figure S2. Calibration plot__

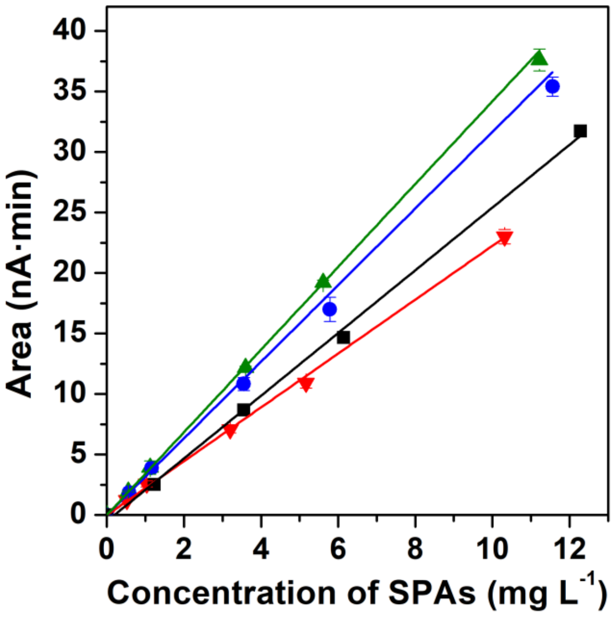

Figure S2. Calibration plot of PG (■), TBHQ (●), BHA (▲) and BHT (▼) using the HPLC method coupled to the PSS/MWCNTs-COOH/SPCE as electrochemical detector at +0.80 V *vs* Ag.

Figure S3. Analysis of the moisturizing cream using DAD

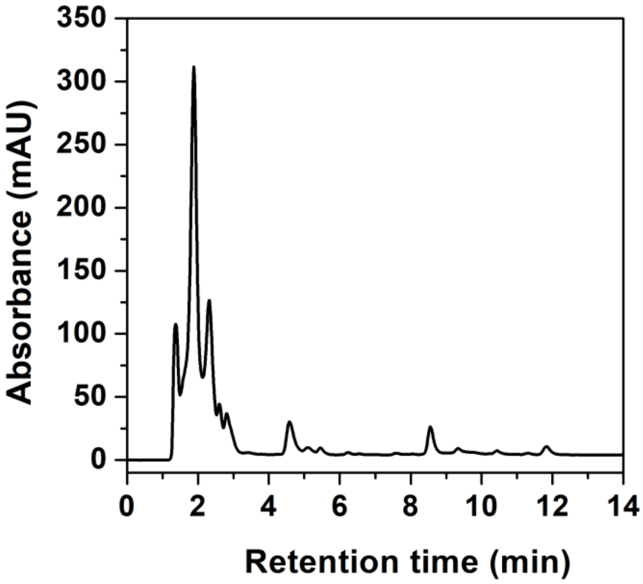

Figure S3. Chromatogram of the moisturizing cream obtained using the diode-array detector at 280 nm.

Table S1. Recovery study for SPA concentration (% w/w) in different cosmetics using the HPLC-ECD method.

Sample	Compound	Concentration (% w/w)	Added (% w/w)	Found (% w/w)	Recovery,%	RSD, %
**Micellar water**	PG	<LOD	0.0020	0.0017	87	5
	TBHQ	<LOD	0.0022	0.0024	109	6
	BHA	<LOD	0.0022	0.0020	93	4
	BHT	0.0018	0.0020	0.0038	99	5
**Moisturizing cream**	PG	*	0.0020	*	*	*
	TBHQ	0.0019	0.0022	0.0037	83	4
	BHA	<LOD	0.0022	0.0019	84	5
	BHT	0.0012	0.0020	0.0031	97	6

* Unable detection
